# Dimensions of caregiver strain may partially mediate the relationship between youth symptomology and counseling utilization among Latinxs

**DOI:** 10.1371/journal.pone.0302575

**Published:** 2024-04-26

**Authors:** Alejandro L. Vázquez, Demi Culianos, Omar G. Gudiño, Cynthia M. Navarro Flores, Tyson S. Barrett, Melanie M. Domenech Rodríguez

**Affiliations:** 1 Department of Psychology, University of Tennessee, Knoxville, Knoxville, Tennessee, United States of America; 2 Department of Psychology, Utah State University, Logan, Utah, United States of America; 3 Department of Psychology, University of Kansas, Lawrance, Kansas, United States of America; 4 Highmark Health, Pittsburgh, Pennsylvania, United States of America; Sreenidhi Institute of Science and Technology, INDIA

## Abstract

Whether Latinx families use youth mental health services (MHS) depends on complex influences of barriers and facilitators within and outside of the home. This research sought to shed light on caregiver strain as part of the equation focused on parental identification and responses to youth mental health needs. We examined multiple dimensions of caregiver strain as potential mediators between youth mental health symptom severity and psychological counseling utilization. The present sample consisted of 598 Latinx caregivers to youths ages 6–18 who provided information on youth internalizing and externalizing problems, caregiver strain, and youth psychological counseling service utilization within the last year. Our findings suggest that youth symptom severity (internalizing and externalizing problems) was generally positively associated with dimensions of caregiver strain. Youth symptom severity through *objective* and *subjective internalized strain* pathways were associated with greater odds of youth MHS utilization. In contrast, youth symptom severity through *subjective externalized strain* reduced the odds that Latinx caregivers would report utilizing youth MHS. These models only partially mediated the relationship between youth problems and service use. Findings suggest that Latinx caregivers may navigate conflicting sources of strain related to their child’s mental health problem severity in ways that may differentially impact the odds that they access youth MHS. Along with addressing structural and systemic barriers to care, utilization of psychological counseling services may also be improved through interventions that help Latinx caregivers view youth services as avenues for addressing caregiver strain and providing psychoeducation that frames externalized strain within a mental health lens.

## Introduction

Latinx youth continue to experience significant disparities in access to mental health services (MHS) despite reforms and policies aiming to improve access, signaling a need to implement outreach and intervention strategies focusing on the cultural and structural needs of Latinx families to improve access to needed MHS [1]. Research has increasingly focused on how culture-bound factors impact Latinx caregivers’ recognition of youth mental health problems and subsequent help-seeking to inform efforts for reducing mental health disparities (e.g., etiological examinations of psychopathology, service utilization preferences, and the impact of acculturation on service utilization patterns; [2–5]). Recent research has suggested that dimensions of caregiver strain among Latinxs, or stress resulting from caring for a youth with emotional and/or behavioral problems are an important culture-bound pathway by which parents become aware of the need for formal youth MHS and decide to seek support [4,6]. However, research has yet to test this assertion regarding mediational pathways between youth mental health problems and MHS utilization through multiple dimensions of caregiver strain among Latinxs.

### Theoretical frameworks

The present study draws from theoretical frameworks that conceptualize caregiver strain as a culturally bound pathway by which parents may become aware of problematic youth behaviors and determine whether seeking MHS is warranted. Brannan and colleagues provide a conceptual framework for understanding the development of caregiver strain stemming from caring for a youth with emotional and/or behavioral problems and subsequent help-seeking behaviors [7]. Youths’ mental health problems and concurrent family stressors pile up and can exceed the caregiver’s ability to cope. Youth symptomology and other life stressors can directly contribute to the development of caregiver strain and subsequent youth MHS utilization as a coping response. Additional mediational pathways include family recourses (i.e., material resources, social supports, psychological well-being, family functioning) and perceptions (i.e., casual attributions, attitudes about treatment, expectations about life, views of family role), that form indirect pathways between stressors and the development of caregiver strain and subsequent service utilization. While caregiver strain is one part of the equation in the identification and responses to youth mental health needs, it is important to note that the utilization of MHS depends on complex influences of barriers and facilitators within and outside of the home.

Brannan and colleagues [8] identified three subtypes of caregiver strain within this model that may differentially impact help-seeking behaviors: objective, subjective internalized, and subjective externalized. According to this conceptualization, objective strain refers to negative experiences associated with caring for a child with emotional and/or behavioral problems (e.g., interruptions to personal time and/or work commitments, financial burden on the family, disruption of family functioning, impact on mental and physical health). Subjective internalized strain refers to feelings that the caregiver has internalized in response to their youth’s emotional and/or behavioral problems (e.g., sadness/unhappiness, concerns regarding the family’s future and/or child’s future, exhaustion). Lastly, subjective externalized strain refers to the caregivers’ negative feelings regarding the child (e.g., anger, resentment, embarrassment, ability to relate to the child). It is imperative to consider these domains of caregiver strain stemming from youth symptomology to improve researchers’ understanding of perceptual factors associated with help-seeking behaviors among Latinx families.

The development of caregiver strain is associated with casual attributions about youth problems, attitudes about mental health treatments, and judgments about the severity of youth problems and the need for MHS, which can vary across cultures [7]. Weisz and colleagues developed a threshold model to understand racial/ethnic differences in caregiver strain stemming from caring for a youth with emotional and/or behavioral problems [9]. Caregivers are important gatekeepers to youth MHS as their odds of seeking help for their youth are influenced by the degree of strain they experience from their child’s emotional and/or behavioral problems. Cultural differences in interpretations of the severity and longevity of youth mental health problems are known to vary between cultural groups, potentially contributing to different thresholds for whether youth problems are of sufficient severity to require formal intervention. Examining the perceptual threshold for help-seeking could improve our understanding of youth problem recognition and subsequent help-seeking behaviors among Latinx caregivers.

### Perceptual differences in mental health problems

Latinx caregivers underutilize youth psychological counseling services despite reporting the clinical need for these services and noting that such services are a preferred source of support for addressing youth mental health problems [2]. These disparities in access may reflect structural barriers to utilizing youth MHS that disproportionally impact people of color like Latinxs [10]. Cultural differences in perceptual thresholds determining whether youth problems are of sufficient severity to warrant formal intervention may also contribute to the underutilization of preferred youth psychological counseling services among Latinx caregivers [11,12]. For example, findings from the Pathways to Latinx Mental Health Study found that Latinx caregivers often reported delaying or avoiding engaging in youth MHS due to perceptions that their child’s problems were typical, despite reporting clinically elevated mental health problems [13]. Thus, while Latinx caregivers report both clinical need and that psychological counseling services are a preferred method of addressing youth psychopathology, the probability that they access these services may be in part influenced by culturally-bound perceptions about whether their youth’s difficulties represent mental health problems and are of sufficient magnitude to warrant formal intervention.

### Relationship between caregiver strain, youth problems, and MHS

Parents often report experiencing caregiver strain in response to the emergence of youth emotional and/or behavioral problems [14,15]. There is a well-documented positive association between youth problem severity and caregiver reported parenting strain [14,16]. The challenges of caring for a child with emotional or behavioral problems may alert caregivers to the severity of their youths’ problems and may also direct them towards seeking formal youth MHS [7]. Indeed, research has suggested that parenting stress related to youth problems is an important predictor of caregivers’ reported need for and utilization of youth MHS [17]. Research has connected these findings through the examination of global caregiver strain as a potential mediator between youth problems and cumulative MHS utilization [4], which may obscure the relationship between different dimensions of caregiver strain that may move Latinx families toward or away from utilizing youth psychological services.

Research on caregiver strain has often focused on global measures of parenting stress that preclude a more nuanced assessment of the impact of caring for a youth with emotional and/or behavioral problems [2,15]. Recent research suggests that Latinx caregivers may develop different subtypes of caregiver strain in response to specific youth problem types. Galvan and Gudiño [6] found that externalizing problems predicted subjective internalized and externalized strain, but not objective strain, among Latinx caregivers. In contrast, youth internalizing problems were associated with greater objective and subjective internalized strain. These findings suggest that the impact of youth internalizing and externalizing problems may place different stressors on Latinx caregivers.

There is little research focused on the association between multiple dimensions of caregiver strain and youth’s actual utilization of MHS. Of note, Brannan and colleagues [7] did examine dimensions of caregiver strain as predictors of patterns of youth MHS utilization. In their primarily White sample, they found that only subjective internalized strain was associated with youth MHS use. Specifically, they found that lower subjective internalizing strain was associated with greater odds of utilizing youth outpatient services, while higher subjective internalizing strain was associated with greater odds of caregivers reporting utilization of both youth outpatient and inpatient services. Shin and Brown [4] examined global caregiver strain as a mediator between caregiver race/ethnicity (i.e., Latinx and Black relative to White) and a latent factor representing utilization of multiple MHS (i.e., specialty inpatient and outpatient, non-specialty outpatient, school-based services). While this research controlled for mental health service need, the analysis primarily focused on understanding racial/ethnic differences in caregiver strain on youth MHS use and did not examine indirect pathways between youth mental health service need and MHS utilization through subtypes of caregiver strain (e.g., objective, subjective internalized, subjective externalized). Little research has examined dimensions of caregiver strain as predictors of youth MHS use among Latinx families. Recent research from the Pathway to Latinx Mental Health Study found that greater objective strain was associated with an increased likelihood that Latinx caregivers would report utilizing youth telepsychology services during the Coronavirus Pandemic [18]. Research has yet to examine subjective internalized and externalized strain as predictors of youth MHS utilization among Latinx caregivers. These findings suggest that dimensions of caregiver strain may have differential impacts on caregivers’ odds of reporting the utilization of youth outpatient MHS that have yet to be explored among Latinxs.

### The present study

Research has highlighted the importance of understanding cultural perceptions of mental health problems and related interventions [19]. Work to date has documented specific associations between youth psychopathology, multiple dimensions of caregiver strain, and their differential impacts on youth MHS utilization outcomes. However, research is needed to understand potential pathways linking these constructs. Examining dimensions of caregiver strain as potential mediators between youth problems and MHS utilization among Latinx caregivers will bridge this gap. The present study aimed to (a) test whether dimensions of caregiver strain (i.e., objective, subjective internalized, subjective externalized) may mediate the relationship between youth problems and utilization of psychological counseling services and (b) determine whether these relationships differed by youth problem type. Based on prior research, we hypothesized that the dimension of caregiver strain would mediate the relationship between youth problems (i.e., internalizing and externalizing) and MHS. Specifically, the effect of youth problems through objective strain was expected to be associated with an increase in the odds that caregivers would report using youth MHS in the last year. Analyses examining the mediating effects of subjective and internalizing strain were considered exploratory due to limited information regarding the potential impact of these factors on Latinx caregivers’ odds of reporting youth MHS use. Tests of whether any observed mediated relationships differed by problem type were also considered exploratory.

## Method

### Procedure

The present study utilized data from 598 Latinx caregivers who participated in the Pathways to Latinx Mental Health study [2]. Participants were recruited online through Qualtrics, an online survey panel company. Research has found that such panels provide data comparable to traditional methods of survey administration (i.e., paper and pencil) when attention checks are used [20]. These checks were a combination of questions built into the survey and software tools that were monitored by the research team and a Qualtrics survey manager. Validation methods included contrasting methods used to identify respondents who provided poor quality data (e.g., directed responses, inconsistent reporting, speeding, straight lining, or zigzagging; [21]).

Qualtrics recruited participants between May 21, 2020 and June 18, 2020. Qualtrics has access to survey panels that include individuals who have registered to be contacted regarding research opportunities. Respondents who could meet inclusion criteria were contacted by Qualtrics via email and were provided a description of the study, required time, and compensation. Those who were interested completed a brief screener to determine their eligibility. Participants who were eligible for the study were asked to read a letter of information describing the purpose of the survey and provided written consent. Participants completed a 20-min online survey assessing MHS utilization, youth problems, and caregiver strain. If caregivers had multiple children, they were asked to report on the child that presented the most challenges to them as a parent. Responses were required for all survey items. Identifying information was not collected as part of the survey. Approval to conduct the present study was obtained from the Utah State University Institutional Review Board (protocol # 11045).

### Participants

Participants met inclusion criteria if they were (a) Latinx, (b) a caregiver to at least one youth between the ages 6–18, (c) able to complete the survey in English, and (d) residing within the United States. Roughly a thousand participants reported that they met inclusion criteria (*n* = 1,128). Participants were excluded when they did not provide informed consent (*n* = 17), provided poor quality data as identified by attention checks (*n* = 235), or did not complete the entire survey (*n* = 278). The final sample consisted of 598 caregivers who were 38.53 years old on average (*SD* = 9.08), predominantly female (*n* = 420; 70.2%), a biological parent (*n* = 565, 94.5%), had higher levels of educational attainment (high school, equivalent, or less *n* = 198, 33.1%; post-secondary *n* = 400, 66.9%), commonly preferred speaking English and Spanish equally (*n* = 271, 45.3%), and were second generation (*n* = 283, 47.3%). The most frequently reported household income was between $30,000-$49,999 (*n* = 129, 21.6%). Youths were 11.87 years old on average (*SD* = 3.36), most frequently boys (*n* = 328, 54.8%), and largely had health insurance (*n* = 576, 96.3%). For additional information regarding this sample see [2,18].

### Measures

#### Demographic characteristics

Caregivers were asked to report their demographic characteristics (i.e., age, binary-gender, academic attainment, generational status, preferred language, household income) and that of their child (i.e., age, binary-gender, insurance status).

#### Emotional and behavioral problems

The Child Behavior Checklist (CBCL) is a 113-item questionnaire that assesses various emotional and behavioral problems for youth ages 6–18 [22]. Caregivers reported on the frequency of each problem for their youth over the last 6 months. Responses are (0) *not true*, (1) *sometimes true*, or (2) *often true*. Composite *t* scores can be calculated from CBCL items to represent youth internalizing (32 items; e.g., depression, anxiety, somatization) and externalizing problems (35 items; e.g., aggression, rule-breaking behavior). Within the present sample, internal consistency for CBCL internalizing (α = .95) and externalizing (α = .96) subscales was excellent.

#### Caregiver strain

The Caregiver Strain Questionnaire (CGSQ) is a 21-item self-report measure that assesses the impact of parenting a youth with emotional or behavioral problems [8]. After conducting a confirmatory factor analysis on the CGSQ in the Pathways to Latinx Mental Health dataset, item 14 of the original measure was not producing the expected positive valance after reverse coding and thus was excluded (“How well did you relate to your child?”). The 20-item CGSQ is composed of three subscales: objective strain (11 items), subjective externalized strain (3 items), and subjective internalized strain (6 items). Caregivers report how much of a problem each statement was in the last year: (1) *not at all*, (2) *a little*, (3) *somewhat*, (4) *quite a bit*, or (5) *very much*. Higher scores on CGSQ subscales represented greater caregiver strain. Within the present sample, internal consistency for objective strain (α = .96), subjective internalized strain (α = .91), and subjective externalized strain subscales (α = .85) ranged from good to excellent.

#### Mental health service utilization

Information regarding the utilization of youth MHS was collected using the Caregiver Support Services Questionnaire (CSSQ; [23]). The CSSQ is a self-report measure that asks caregivers to report on their utilization of 11 youth support services (i.e., “*In the last year*, *did your child receive help from any of the following support services*?”) and queries whether each source of support was utilized within the last year. The present study used a single item from the CSSQ that asked caregivers to report their use of psychological counseling or therapy for their youth in the last year (e.g., “*Psychological counseling or therapy*?”). Responses were *yes* or *no*. The CSSQ has been previously used to examine youth MHS utilization patterns and related correlates among Latinx families who participated in the Pathways to Latinx Mental Health study [2,18].

### Analytic plan

Statistical analyses were performed using R version 4.0.5 [24] in the RStudio statistical environment [25], with the marginalmediation [26], margins [27], psych [28], tidyverse [29], and furniture [30] packages. Kruskal-Wallis rank sum tests were used to determine whether youth age, caregiver strain subscales, and CBCL composite scores (i.e., internalizing, externalizing) significantly differed between caregivers of youth who utilized psychological counseling services in the last year and those who did not. A chi-square test of independence was used to determine whether psychological counseling utilization differed by youth binary-gender. Kendall’s Tau bivariate correlations were used to examine associations between youth demographics (i.e., age, binary-gender), problems (i.e., internalizing and externalizing T scores), and dimensions of caregiver strain (i.e., objective, subjective externalizing, subjective internalizing). Mediation analyses were then conducted independently for internalizing and externalizing problems using the Marginal Mediation framework [31]. Marginal Mediation was used as it allowed for the estimation of mediated effects with mixed variable/outcome types (i.e., continuous and dichotomous). A-paths were estimated using a generalized linear model with a Gaussian distribution and an identity link. B and C paths were estimated using a generalized linear model with a binomial distribution and logit link (i.e., logistic regression). Average marginal effects (AME) were used to convert estimates from logistic regression models into effect sizes in linear units. AMEs represent absolute change in probability, while other common effect sizes such as odds ratios represent relative change in odds. Indirect effects were calculated by multiplying A and B paths [32]. Confidence intervals (CI) were estimated using bootstrapping with 1,000 bootstrapped samples. Effect sizes were also calculated using standardized variables to facilitate comparisons.

## Results

Within the present sample, caregivers’ average *t* scores on the CBCL were 54.69 (*SD* = 14.69) for internalizing problems and 52.29 (*SD* = 13.79) for externalizing problems. Average caregiver strain scores were highest for the subjective internalized subscale (*M* = 2.10, *SD* = 1.09), followed by objective (*M* = 1.71, *SD* = 0.93) and subjective externalized subscales (*M* = 1.61, *SD* = 0.91). CBCL *t* scores and caregiver strain subscales scores were significantly higher among youths who utilized psychological counseling in the last year. Caregivers were significantly more likely to report utilizing psychological counseling for boys relative to girls. Psychological counseling did not significantly differ by youth age. See Table 1 for youth demographics, problems, and caregiver strain by psychological counseling utilization. There were also significant positive correlations between both types of youth problems and all three dimensions of caregiver strain. Youth age was not significantly associated with youth problems or dimensions of caregiver strain. Youth binary-gender was positively associated with internalizing problems, with boys having higher problem severity. See Table 2 for the bivariate correlation matrix. Given its association with caregiver reported internalizing problems and psychological counseling utilization, youth binary-gender was included as a covariate in the mediation models. As youth age was not significantly associated with internalizing and externalizing problems or service utilization, it was not included as a covariate in mediation analyses.

**Table 1:** Youth demographics, problems, and caregiver strain dimensions by service utilization outcome.

		Psychological counseling		
	Total	Yes	No	*p* value
Mean/SD (Frequency/%)	*N* = 598	*n* = 210	*n* = 388	
Youth age	11.87 (3.36)	11.83 (3.36)	11.89 (3.37)	.839
Youth binary-gender				.034
Boys	328 (54.8%)	128 (61%)	200 (51.5%)	
Girls	270 (45.2%)	82 (39%)	188 (48.5%)	
Internalizing problems	54.69 (14.69)	64.98 (12.66)	49.12 (12.55)	< .001
Externalizing problems	52.29 (13.79)	61.15 (12.06)	47.50 (12.22)	< .001
Objective strain	1.71 (0.93)	2.35 (0.97)	1.36 (0.68)	< .001
Subjective internalized strain	2.10 (1.09)	2.84 (1.02)	1.70 (0.91)	< .001
Subjective externalized strain	1.61 (0.91)	2.07 (1.06)	1.37 (0.71)	< .001

Note: p values for continuous variables reflect significant Kruskal-Wallis rank sum test. *P* value for youth binary-gender reflects chi-square test of independence.

**Table 2:** Correlations between youth problems and caregiver strain dimensions (*N* = 598).

	[1]	[2]	[3]	[4]	[5]	[6]	[7]
[1] Youth age	1						
[2] Youth binary-gender ^a^	-.05	1					
[3] Internalizing problems	.00	.08*	1				
[4] Externalizing problems	-.05	.06	.59***	1			
[5] Objective strain	-.05	.07	.55***	.60***	1		
[6] Subjective internalized strain	-.03	.07	.51***	.53***	.66***	1	
[7] Subjective externalized strain	-.07	.06	.42***	.51***	.62***	.57***	1

Note:

*** *p* < .001,

** *p* < .001,

* *p* < .05.

Estimates represent Kendal’s Tau.

^a^ (1) boys relative to (0) girls.

### Mediation: Direct effects

**Fig 1:**
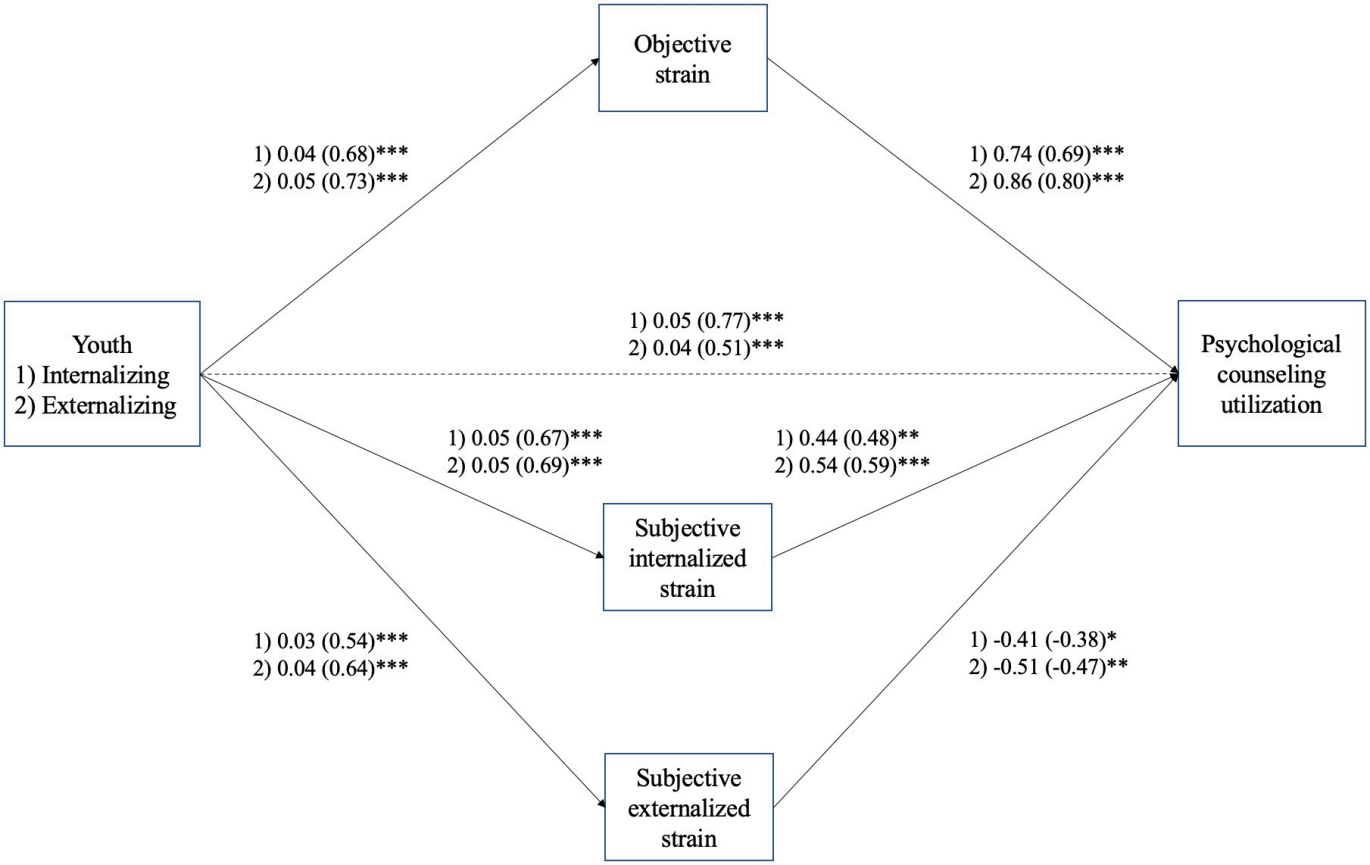
Mediation models (*N* = 598). This figure shows the results of mediation models examining individual paths regarding youth problem type and psychological counseling utilization *** *p* < .001, ** *p* < .01, * *p* < .05. Unstandardized(standardized) estimate. The outcome is not standardized as it is binary. Estimates reported control for youth binary-gender.

There was a statistically significant direct effect between internalizing problems and the psychological counseling outcome. For a one unit increase in internalizing problems, there was an associated 0.12 (Standardized AME, CI [0.07, 0.16]; Unstandardized AME = 0.008, CI [0.005, 0.011]) increase in the probability of psychological counseling utilization, when controlling for objective strain, subjective internalized strain, subjective externalized strain, and youth binary-gender. See Fig 1 for caregiver strain pathways between youth problems and psychological counseling utilization. There was also a significant direct effect between externalizing problems and the psychological counseling outcome. For a one unit increase in externalizing problems, there was an associated 0.08 increase (Standardized AME, CI [0.04, 0.12]; Unstandardized AME = 0.006, CI [0.003, 0.009]) in the probability of psychological counseling utilization, when controlling for objective strain, subjective internalized strain, subjective externalized strain, and youth binary-gender.

### Mediation: Indirect effects

**Fig 2:**
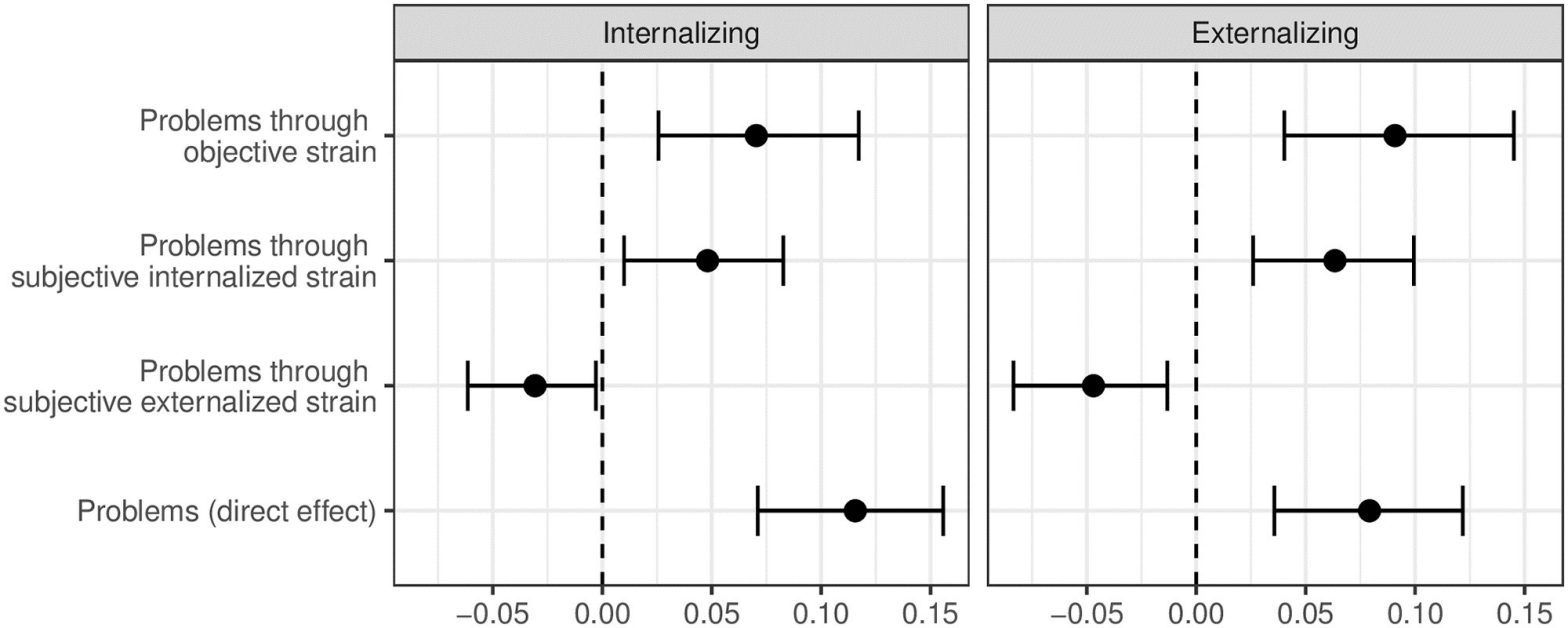
Probability of psychological counseling (*N* = 598). This figure shows the comparison of standardized indirect and direct effects on the probability of receiving psychological counseling. Results are organized by youth problem type. Estimates reported control for youth binary-gender.

There was a statistically significant effect between internalizing problems and psychological counseling utilization through objective strain. For a one unit increase in internalizing problems, through its effect on objective strain, there was an associated 0.07 (Standardized AME, CI [0.03–0.12]; Unstandardized AME = 0.005, CI [0.002, 0.008]) increase in the probability of psychological counseling utilization. For a one unit increase in internalizing problems, through its effect on subjective internalized strain, there was an associated 0.05 (Standardized AME, CI [0.01, 0.08]; Unstandardized AME = 0.003, CI [0.001, 0.006]) increase in the probability of psychological counseling utilization occurring. For a one unit increase in internalizing problems, through its effect on subjective externalized strain, there was an associated -0.03 (Standardized AME, CI [-0.06, -0.003]; Unstandardized AME = -0.002, CI [-0.004, -0.000]) decrease in the probability of utilizing psychological counseling. Dimensions of caregiver strain did not fully mediate the relationship between youth internalizing symptom severity and MHS utilization. A comparison between indirect effects for the internalizing problems model is presented in Fig 2.

Significant caregiver strain pathways were also found between youth externalizing problems and psychological counseling utilization. For a one unit increase in externalizing problems, through its effect on objective strain, there was an associated 0.09 (Standardized AME, CI [0.04, 0.15]; Unstandardized AME = 0.007, CI [0.003, 0.011]) increase in the probability of psychological counseling utilization. For a one unit increase in externalizing problems, through its effect on subjective internalized strain, there was an associated 0.06 (Standardized AME, CI [0.03, 0.10]; Unstandardized AME = 0.005, CI [0.002, 0.007]) increase in the probability of reporting utilization of psychological counseling. For a one unit increase in externalizing problems, through its effect on subjective externalized strain, there was an associated -0.05 (Standardized AME, CI [-0.08, -0.01]; Unstandardized AME = -0.003, CI [-0.006, -0.001]) decrease in the probability of psychological counseling utilization occurring. Dimensions of caregiver strain did not fully mediate the relationship between externalizing symptom severity and MHS utilization. A comparison between indirect effects for the externalizing problems model is presented in Fig 2.

## Discussion

The present study represents a step towards understanding factors contributing to Latinx caregivers’ utilization of youth MHS. Our findings suggest that youth symptom severity is associated with greater caregiver strain across all dimensions, regardless of problem type (i.e., internalizing vs. externalizing problems). However, only the effects of youth symptom severity through *objective* and *subjective internalized strain* pathways were associated with greater odds of youth MHS utilization. Youth symptom severity through *subjective externalized strain* reduced the odds that Latinx caregivers would report utilizing MHS for their child. While these pathways were significant, they only partially mediated the relationship between youth problems and MHS utilization in the last year. As conceptualized in this study, caregiver strain is just one predictor that does not fully account for MHS utilization patterns among Latinx families. Nonetheless, these findings highlight the importance of examining a range of mechanisms as effective interventions for eliminating disparities will need to target relevant mechanisms.

Results from mediation analysis suggest that some dimensions of caregiver strain (i.e., objective and subjective internalized) are associated with an increased probability that caregivers will access MHS. This suggests that observable consequences (e.g., missing work) associated with caring for a youth with emotional and/or behavioral problems and the negative emotional or psychological impact on the caregiver may be particularly important drivers of MHS utilization. Caregivers experiencing objective and/or subjective internalized strain may view youth MHS as an appropriate method of reducing child symptom severity and associated impact on caregivers, thus increasing their chances of seeking formal services [17].

Our findings further suggest that subjective externalized strain may be a pathway through which Latinx caregivers may be *less* likely to utilize youth MHS. Such responses would include things like being angry at the youth or embarrassed about the youth’s difficulties. It is possible that Latinx caregivers experiencing subjective externalized strain in response to their youth’s emotional and/or behavioral challenges may not view these challenges as “mental health” or may therefore not view MHS as appropriate. Instead, other avenues for responding to the challenges may be pursued. For example, it is conceivable that Latinx caregivers experiencing elevated subjective externalized strain may attribute their child’s problems to innate characteristics not amenable to therapy or may instead resort to harsh parenting in place of seeking formal services [33]. Caregivers may fear social services involvement if harsh parenting is detected by a provider [34]. Alternatively, caregivers who are embarrassed about their youth’s difficulties or their own behavior in response to these problems may be unlikely to seek formal services for these problems. Embarrassment and service utilization have been connected in a sample of caregivers of medically vulnerable adults [35]. Further research is needed to unpack this mediated pathway to improve the chances that Latinx caregivers experiencing elevated subjective externalized strain in response to youth mental health symptoms will access MHS for their youth and experience decreased caregiver strain themselves. For example, researchers may consider examining family resources (e.g., material resources, social supports, family functioning) and perceptions (e.g., casual attributions, attitudes about MHS) that contribute to both the development of caregiver strain and the use of youth MHS as a coping response [7].

In contrast to prior research, youth mental health symptoms (i.e., externalizing and internalizing) were significantly associated with all dimensions of caregiver strain [6]. These findings may be attributed to potential differences in caregiver acculturation, which is known to influence interpretations of youth mental health problems [3]. The sample used by Galvan and Gudiño [6] included greater representation from caregivers who were first generation in the United States, while the present sample was predominately bicultural (i.e., 45.3% equal preference for English or Spanish) and second generation in the United States (47.3%). As perceptual thresholds regarding the severity of youth mental health problems and resulting caregiver strain may vary by acculturation level, future research should control for caregiver acculturation level when examining mediational models of youth MHS utilization among Latinxs. Furthermore, the sample used by Galvan and Gudiño [6] also included families with a high likelihood of seeking services (i.e., families were known to public service systems), whereas the present sample was a community sample. It is possible that dimensions of caregiver strain might be important when teasing apart high service users but might be less relevant when predicting MHS in a general sample. Future research may test our mediation model within a sample of high service users.

Our findings should not be misconstrued as presenting dimensions of caregiver strain as the only pathways by which youths can become engaged in MHS. Indeed, our results show that these factors only partially mediated the relationship between youth mental health symptom severity and MHS utilization, which does not support such a narrow focus on the mechanisms underpinning help-seeking behaviors among Latinx caregivers. While caregiver strain may be one important factor influencing caregiver perceptions of youth needs, individual youth factors, family factors, and systemic issues outside of the family’s control also influence rates of MHS utilization. As a result, our findings should be viewed as a step toward understanding Latinx caregivers’ reactions to youth problems and help-seeking within a broader context that erects structural barriers to care. A comprehensive understanding of factors influencing MHS utilization is needed, as efforts to eliminate disparities will need to target relevant mechanisms. Indeed, a combination of efforts to eliminate structural, cultural, familial, and individual barriers to MHS utilization is warranted. These results focus on one factor at the interface of the youth and caregiver, which may be particularly influential for mobilizing caregiver responses. Further research is needed to confirm our findings using longitudinal data to ensure temporal precedence between predictors, mediators, and the outcome [36]. Additional research may also extend our findings by examining whether the mediating influence of dimensions of caregiver strain may vary depending on the service type [7].

### Implications

Despite the need for equitable access to youth MHS, Latinx youth often face disproportionate barriers to accessing care. Utilization of MHS depends on complex influences of barriers and facilitators within and outside of the home. This research sheds light on one specific part of the equation focused on caregiver identification and responses to youth mental health needs. As found more broadly across the literature, we find that caregiver strain is also one important consideration when predicting whether Latinx caregivers will seek MHS for their youth. In particular, objective consequences and feelings of sadness or worry about a youth’s difficulties emerged as important drivers of MHS utilization. Conversely, feelings of anger, resentment, or embarrassment about a youth’s difficulties were associated with a decreased likelihood of MHS use. Along with addressing structural and systemic barriers outside of the home, utilization of psychological counseling services may also be improved through interventions that (a) help caregivers view youth MHS as avenues for addressing caregiver strain too and (b) education to help caregivers experiencing externalized strain understand their strain through a mental health lens.

### Limitations

The present study has several limitations. While the literature documents caregiver strain emerging in response to youth problems impacting the chances that families will use youth MHS, temporal precedence between predictors, mediators, and the outcome cannot be established with the present cross-sectional design. While our proposed mediators were measured as strain resulting from youth problems, future research should confirm our findings with repeated longitudinal measures that ensure the temporal order of variables within the mediation model [36]. Future research should consider using ecological momentary assessments to examine the interplay between youth psychopathology, caregiver strain, and help-seeking attitudes across time in naturalistic settings. The present study used caregiver-reported data. As youth MHS access is influenced by the willingness of the child to engage in services, future research should gather data from youths to better understand interpersonal factors contributing to MHS access within this group. Data for the present study focused on caregivers who could complete the survey in English due to feasibility and cost issues associated with recruiting Spanish speaking caregivers through Qualtrics survey panels. This may have contributed to a sampling of more acculturated Latinx caregivers. As acculturation is known to impact perceptions of what constitutes a mental health problem [3], future research should administer the survey in English and Spanish and assess caregiver acculturation level. Despite these limitations, the present study represents a step toward understanding factors impacting youth MHS utilization among an understudied Latinx subpopulation.

## Conclusions

Our findings suggest that Latinx caregivers may navigate conflicting sources of strain related to their child’s mental health problem severity that may differentially impact the odds that they may access MHS for their youth. Youth symptom severity through *objective* and *subjective internalized strain* pathways appear to be associated with greater odds of caregivers reporting utilization of youth MHS. In contrast, youth symptom severity through *subjective externalized strain* may reduce the odds that Latinx caregivers would report utilizing MHS for their child. These findings highlight the importance of investigating dimensions of caregiver strain alongside systemic factors contributing to youth MHS disparities among Latinx families. Despite these promising findings, further research is needed to confirm these findings using longitudinal data that ensures temporal precedence between predictors, mediators, and the outcome.

## Supporting information

S1 Data (CSV)
